# Designing Electrochemical Biosensing Platforms Using Layered Carbon-Stabilized Porous Silicon Nanostructures

**DOI:** 10.1021/acsami.2c02113

**Published:** 2022-03-14

**Authors:** Keying Guo, Maria Alba, Grace Pei Chin, Ziqiu Tong, Bin Guan, Michael J. Sailor, Nicolas H. Voelcker, Beatriz Prieto-Simón

**Affiliations:** † Monash Institute of Pharmaceutical Sciences, Monash University, Parkville, Victoria 3052, Australia; ‡ Melbourne Centre for Nanofabrication, Victorian Node of the Australian National Fabrication Facility, Clayton, Victoria 3168, Australia; § Commonwealth Scientific and Industrial Research Organisation (CSIRO), Clayton, Victoria 3168, Australia; ∥ Future Industries Institute, University of South Australia, Mawson Lakes, South Australia 5095, Australia; ⊥ Department of Chemistry and Biochemistry and Department of Nanoengineering, University of California, San Diego, La Jolla, California 92093-0358, United States; ∇ Department of Electronic Engineering, Universitat Rovira i Virgili, Tarragona 43007, Spain; ○ ICREA, Pg. Lluís Companys 23, Barcelona 08010, Spain

**Keywords:** porous silicon, layered nanostructures, controllable surface chemistry, dual-surface functionality, electrochemical biosensor

## Abstract

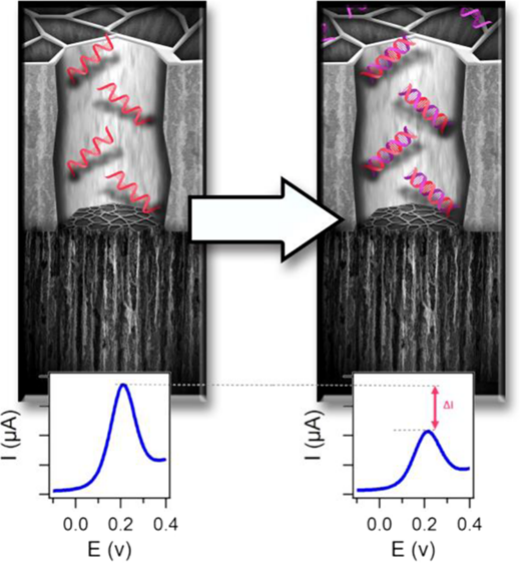

Porous silicon (pSi) is an established porous material that offers ample opportunities for biosensor design thanks to its tunable structure, versatile surface chemistry, and large surface area. Nonetheless, its potential for electrochemical sensing is relatively unexplored. This study investigates layered carbon-stabilized pSi nanostructures with site-specific functionalities as an electrochemical biosensor. A double-layer nanostructure combining a top hydrophilic layer of thermally carbonized pSi (TCpSi) and a bottom hydrophobic layer of thermally hydrocarbonized pSi (THCpSi) is prepared. The modified layers are formed in a stepwise process, involving first an electrochemical anodization step to generate a porous layer with precisely defined pore morphological features, followed by deposition of a thin thermally carbonized coating on the pore walls via temperature-controlled acetylene decomposition. The second layer is then generated beneath the first by following the same two-step process, but the acetylene decomposition conditions are adjusted to deposit a thermally hydrocarbonized coating. The double-layer platform features excellent electrochemical properties such as fast electron-transfer kinetics, which underpin the performance of a TCpSi-THCpSi voltammetric DNA sensor. The biosensor targets a 28-nucleotide single-stranded DNA sequence with a detection limit of 0.4 pM, two orders of magnitude lower than the values reported to date by any other pSi-based electrochemical DNA sensor.

## Introduction

Porous silicon (pSi) has demonstrated its advantages and versatility in various biomedical applications, such as biosensing and drug delivery.^1−4^ The ease of controlling pore morphology has implications in its sensing capabilities and has engendered advances in the detection of a large range of chemical and biological species.^5,6^ Other features of pSi, such as the large available internal surface area and excellent biocompatibility, have also been harnessed to develop high-performance biosensing platforms.^7,8^ Of particular interest for biosensing purposes is the versatile surface chemistry of pSi, allowing a broad range of functionalization routes (e.g., hydrosilylation,^9−11^ silanization,^12^ carbon-based thin layers^13,14^) to introduce functional groups further used to covalently immobilize diverse biomolecules as bioreceptors (e.g., antibodies,^15,16^ oligonucleotides,^17,18^ enzymes^19,20^), and simultaneously protect from surface degradation even under oxidizing conditions.

The advantages of using layered pSi nanostructures as optical sensing platforms have been well demonstrated.^21,22^ Interferometric biosensors based on pSi double layers have been used not only to separate biomolecules based on size exclusion but also to effectively monitor biomolecule penetration into specific layers and improve the sensitivity of the label-free output signal.^19,22^ Control of surface functionalization for each porous layer separately enables discrimination on the basis of molecular affinity via the specific capture probes grafted onto selected layers.

**Figure 1:**
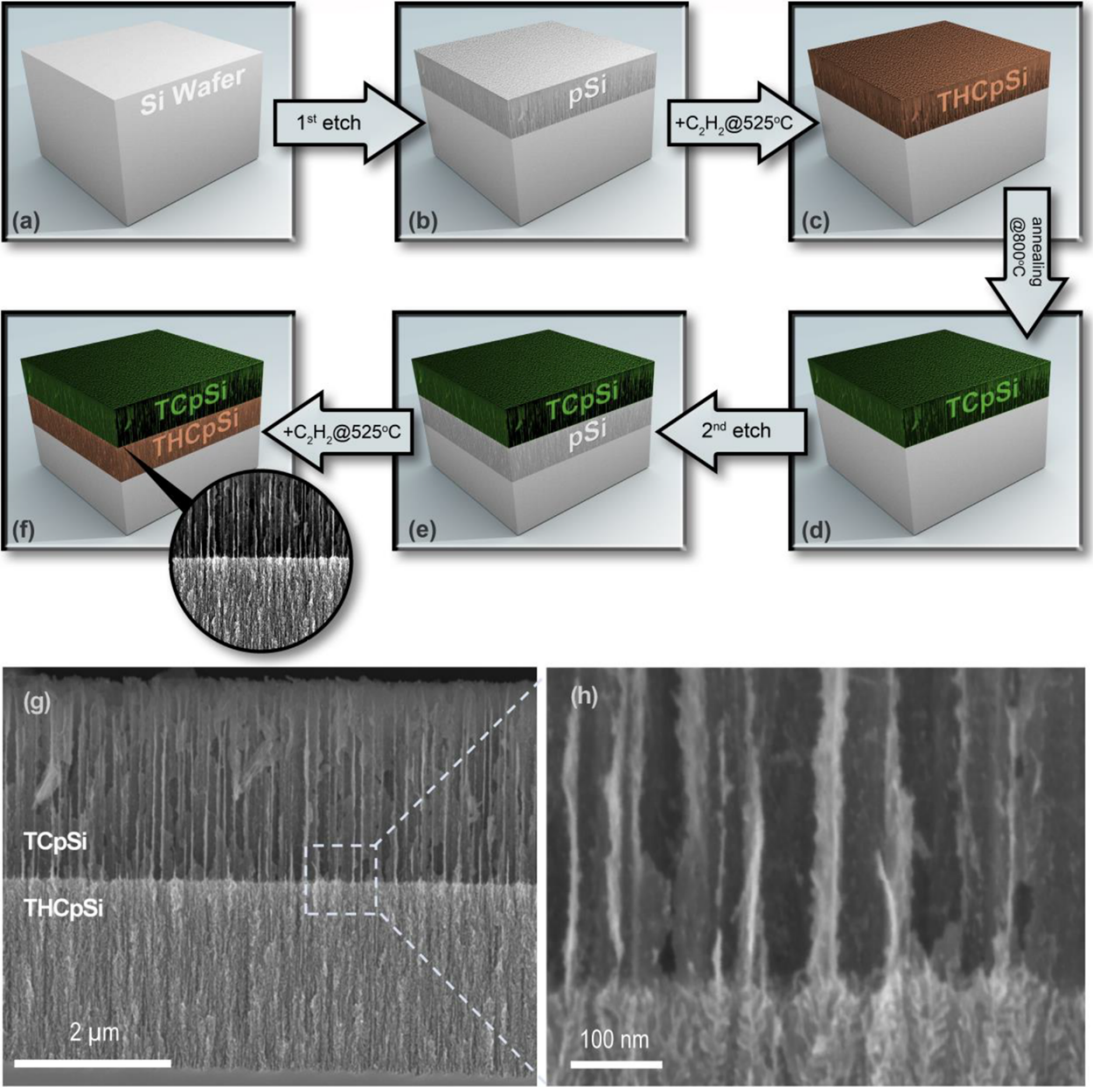
Synthesis of the TCpSi-THCpSi double-layer nanostructure. (a) Bulk silicon wafer. (b) A single layer of porous silicon (pSi) is prepared via electrochemical anodization; this layer is referred to in this work as the top layer. (c) Decomposition of acetylene gas on the pSi surface at 525 °C (thermal hydrocarbonization) followed by (d) annealing at 800 °C (thermal carbonization) generates a TCpSi surface coating on the top pSi layer. (e) A second pSi layer is generated directly underneath the TCpSi top layer via a second anodization step; this layer is referred to as the bottom layer. (f) A second thermal hydrocarbonization at 525 °C of the sample generates a THCpSi surface on the freshly etched bottom layer. Cross-sectional scanning electron microscope (SEM) images of the resulting TCpSi-THCpSi double layer at (g) low magnification and (h) high magnification.

Nonetheless, to the best of our knowledge, no report has yet described the use of layered pSi nanostructures as electrochemical biosensing platforms. This is likely due to the low stability of porous silicon nanostructures when subjected to aqueous electrochemical conditions and the tendency of silicon to form an electrically insulating oxide (SiO_2_). Moreover, the distribution of the space-charge layer within the silicon walls around pore voids favors the restriction of charge transfer to the pore tips. To overcome these limitations and thus allow charge-transfer reactions proceeding over the entire pSi contour, researchers have demonstrated the possibility to prevent oxidation by chemically derivatizing pSi with nonpolar Si–C=C–R linkages^11^ or introducing a protective thin layer of graphene oxide.^13^ These approaches have demonstrated the possibility of pSi to operate in aqueous environments under oxidizing conditions. Similarly, our research group recently explored the use of a carbon-rich coating for the inner pore walls of a single-layer pSi structure, finding that the layer imparted substantial improvements in chemical stability and electrical conductivity, which resulted in an excellent performance as an electrochemical transducer.^14^ Here we combine this carbonization chemistry with the precise control that electrochemical anodization provides in the silicon system, generating a porous double-layer structure that substantially improves the selectivity of the electrochemical system. The double-layer structure combines a top hydrophilic layer of thermally carbonized pSi (TCpSi) and a bottom hydrophobic layer of thermally hydrocarbonized pSi (THCpSi), which is fabricated via a tandem electrochemical anodization/carbonization process (Figure 1a–f). The resulting double layer displays different average pore sizes and surface chemistries in each layer, which enables the discrimination of analyte on the basis of both size and chemical properties. Most importantly for the intended voltammetric DNA sensor, the carbonization chemistry imparts improved stability to the electrochemical biosensor. We report the specific detection of a 28-nucleotide single-stranded DNA (ssDNA) sequence, with a limit of detection (LOD) of 0.4 pM, which is two orders of magnitude lower than the best LOD (50 pM)^23^ achieved for previously reported pSi-based electrochemical DNA sensors.

## Experimental Section

### Materials

p-type Si wafers with 0.00055–0.001 Ω cm resistivity, (100)-oriented were purchased from Siltronix (France). Hydrofluoric acid (HF) (48%, AR grade) was purchased from Scharlau (Australia). Potassium ferrocyanide (K_4_[Fe(CN)_6_]), potassium ferricyanide (K_3_[Fe(CN)_6_]), undecylenic acid, *N*-hydroxysuccinimide (NHS), *N*(3-dimethylaminopropyl) *N*-ethylcarbodiimide hydrochloride (EDC), phosphate-buffered saline (PBS) tablets, 2-(*N*-morpholino)-ethanesulfonic acid (MES), (3-aminopropyl) triethoxysilane (APTES), (3-glycidylxypropyl) trimethoxysilane (GPTMS), sodium chloride, tris(hydroxymethyl)aminomethane, hydrochloric acid (37%), and fluorescein isothiocyanate (FITC) were purchased from Sigma-Aldrich (Australia). Cyanine5 amine (Cy5-NH_2_) was purchased from Luminoprobe (USA). The acetylene gas cylinder (1 m^3^ industrial grade, dissolved) was purchased from BOC (Australia). All the DNA strands were purchased from Integrated DNA Technologies, Inc. The sequence of the amino-modified ssDNA capture probe was 5′-/5AmMC6/GTC CAC GCC GTA AAC GAT GTC GAC TTG G-3′. The amino-modified nonspecific ssDNA capture probe was 5′-/5AmMC6/CAC AAA TTC GGT TCT ACA GGG TA-3′. The sequence of the target ssDNA was 5′-CCA AGT CGA CAT CGT TTA CGG CGT GGA C-3′.

### Apparatus

Scanning electron microscope (SEM) images were obtained with an FEI NovaNano SEM 430 at an accelerating voltage of 10 kV. Attenuated total reflectance Fourier transform infrared (ATR-FTIR) spectroscopy was performed with a Thermo Scientific Nicolet 6700 FTIR spectrometer. Raman spectra were acquired using a Renishaw inVia Raman microscope with a 100 mW 532 nm laser excitation source. A 10% excitation power density was applied to avoid damage to the surface. Fluorescence microscopy images were collected with a laser scanning confocal microscope (Nikon Instrument TIRF with Ti-U system).

### Fabrication of pSi Single Layer

A whole 6-in. p-type Si wafer was anodically etched in an electrolyte solution containing 1:1 (v:v) aqueous 48% HF:absolute ethanol (caution: HF is highly toxic and corrosive, and proper care should be exerted to avoid contact with skin, eyes or lungs) to produce a first pSi layer, using a wet etching system (A.M.M.T. GmbH, Germany), which can load a 6-in. Si wafer with an exposed etching area of 132 cm^2^. Firstly, a sacrificial layer was produced at an anodic current density of 60.6 mA cm^–2^ for 30 s. This was removed with 1 M sodium hydroxide.^24^ The etching cell was rinsed with water followed by absolute ethanol and dried with N_2_ gas. Next, the relevant current density was applied to fabricate the porous layer with the desired pore size. A current density of 18.9 mA cm^–2^ was applied to the etching cell to form pores of 27 ± 9 nm diameter. The freshly etched pSi was finally rinsed with ethanol and stored in a desiccator until further use.

#### Thermal Carbonization of pSi Single Layer (TCpSi)

The freshly etched pSi substrates were cut into 1.5 cm × 1.5 cm pieces and stabilized using the thermal carbonization (TC) treatment with acetylene decomposition described by Salonen et al.^25,26^ TC is a two-step carbonization process that starts with the thermal hydrocarbonization (THC) treatment at 525 °C followed by annealing at 800 °C. For the THC step, the freshly etched pSi was placed into a quartz tube under N_2_ flow at 2 L min^–1^ for 45 min at room temperature. A 1:1 N_2_/acetylene mixture flow was introduced into the tube at room temperature for 15 min after the purging step, then the quartz tube was placed into a preheated tube furnace at 525 °C for another 15 min under the continuous mixture flow. After the THC process, the tube was left to cool down under N_2_ flow. For the second step of TC, without opening the quartz tube, a mixture of 1:1 N_2_-acetylene was flown for 10 min at room temperature, followed by annealing at 800 °C for 10 min only under N_2_ flow (2 L min^–1^). Finally, the tube was left to cool back to room temperature under N_2_ flow.

### Fabrication of pSi Double Layer

A second etching step was applied on the carbon-stabilized TCpSi single layer using a small cell with a 1.5-cm internal diameter O-ring (the etch cell design can be found in the book Porous Silicon in Practice^27^). Each TCpSi substrate was anodically etched in 3:1 (v:v) HF/ethanol by applying a specific current density to form a bottom layer with desired pore sizes (smaller than for the top layer).

#### Thermal Hydrocarbonization of the Freshly Etched pSi Bottom Layer (THCpSi)

The freshly etched pSi bottom layer within the double-layer structure was thermally hydrocarbonized following the THC step previously described in the Thermal Carbonization of pSi Single Layer section.

### Ellipsometry Analysis of THC- and TC-Treated Flat Si Surfaces

The thickness of the carbon layer was measured using a variable angle spectroscopic ellipsometer (J.A. Woollam, USA). Measurements were taken at three different angles per sample (60, 65, and 70°) with an acquisition time of 20 s at each angle. Analysis of samples was performed using the CompleteEASE software. The control sample (HF-rinsed Si surface, Si/HF) was fitted using the standard model for Si with native oxide. The thickness of the native oxide layer on this control sample (Si/HF) was estimated as 0.69 nm. This value was subtracted from the thickness calculations of the carbon layer on THC- and TC-treated Si surfaces. In addition to the native oxide layer, a B-spline layer developed in CompleteEASE was applied to determine the thickness of the carbon layer formed.

### Layering Surface Functionalities on TCpSi-THCpSi Double Layer

Differential functionalization of the TC top layer and THC bottom layer of the prepared TCpSi-THCpSi nanostructures was performed. Firstly, in order to functionalize the THCpSi bottom layer with COOH groups, the double-layer sample was immersed into pure undecylenic acid at 150 °C for 10 h under an inert atmosphere (N_2_). After cooling down to room temperature, the sample was rinsed with absolute ethanol. The TCpSi top layer is natively covered with a thin oxide layer, preventing from potential introduction of −COOH groups as a result of the alkene grafting process. Prior to further reaction, the COOH-modified sample was immersed into 1:1 (v:v) aqueous 48% HF:absolute ethanol solution for 15 min at room temperature and then vacuum filtered from the solution and finally dried at 65 °C for 3 h.^25^ The COOH-terminated THCpSi bottom layer resisted HF attack and was stable until further modification. However, exposure to HF enables hydroxylation of the TCpSi surface. The OH-terminated TCpSi top layer was subsequently modified in 10 mL 0.5% APTES in anhydrous toluene for 30 min at room temperature. The silane solution was then removed and replaced by anhydrous toluene. Then, the sample was sonicated for 3 min in successive washing steps with fresh toluene, 1:1 (v:v) toluene/methanol, methanol, and ethanol, to remove any traces of loosely bound APTES. The sample was dried at 80 °C overnight. To characterize the layered functionalities, the next steps focused on binding two different fluorophores to the COOH-terminated THCpSi bottom layer and NH_2_-terminated TCpSi top layer. The −COOH groups at the THCpSi bottom layer were activated by incubating the functionalized substrates in 10 mg mL^–1^ EDC and 15 mg mL^–1^ NHS in 0.1 M MES buffer, pH 5.5, at room temperature for 30 min to produce succinimidyl ester groups. Subsequently, a 10 μg mL^–1^ Cy5-NH_2_ solution in 10 mM PBS was incubated on the activated surface for 30 min. The Cy5-modified sample was finally rinsed with PBS and absolute ethanol. To modify the NH_2_-terminated TCpSi top layer, a 10 μg mL^–1^ FITC solution in 10 mM PBS was incubated onto the surface for 30 min. The FITC-modified sample was rinsed with PBS and then absolute ethanol. Control samples exclusively modified either with FITC or Cy5 were simultaneously prepared.

### Optical Characterization of pSi Double Layer

Interferometric reflectance spectra were collected using a tungsten lamp (Ocean Optics) and a CCD spectrometer (Ocean Optics S-2000). White light was fed through one end of a bifurcated fiber optic cable and focused through a lens onto the pSi surface with a spot size of approximately 1 mm in diameter. Light reflected from the pSi layer was collected through the same optical lens and transferred to the CCD spectrometer via the second arm of the bifurcated optic cable. A fast Fourier transform using algorithm from the WaveMetrics Inc. Igor program library was applied to the resulting spectra.

### Electrochemical Characterization

Electrochemical measurements were carried out on an electrochemical analyzer (CH Instruments, model 600D series, USA) using a three-electrode configuration in a Teflon cell containing the pSi nanostructure on an aluminum film as the working electrode, a Ag/AgCl reference electrode, and a platinum wire as a counter electrode.

### DNA Detection Using a TCpSi-THCpSi Double Layer-Based Sensor

#### Fabrication and Modification of DNA Sensor

In order to fine-tune the pore size to enable DNA hybridization, the pSi top layer was electrochemically etched in a 1:1 (v:v) aqueous 48% HF:absolute ethanol solution, by applying a current density of 18.9 mA cm^–2^ for 80 s. The pSi bottom layer was etched in a 3:1 (v:v) aqueous 48% HF:absolute ethanol solution, using a Teflon etching cell and applying a current density of 21.4 mA cm^–2^ for 60, 150, and 300 s to obtain samples with different bottom layer thickness. First, in order to form hydroxyl groups on the TCpSi layer, the TCpSi-THCpSi double layer was treated in 1:1 (v:v) aqueous 48% HF:absolute ethanol solution for 15 min and dried at 65 °C for 3 h. Second, the OH-terminated TCpSi top layer was subsequently modified using 10 mL 10% GPTMS dissolved in anhydrous toluene for 30 min at room temperature. After reaction, the samples were thoroughly rinsed with 1:1 (v:v) anhydrous toluene/methanol, pure methanol, and then pure ethanol. Third, 100 μL of either 10 μM of NH_2_-ssDNA capture probe or nonspecific NH_2_-ssDNA capture probe, both prepared in 0.1 M MES buffer, pH 5.5, were incubated on the double-layer nanostructures for 2 h at room temperature. Samples were subsequently rinsed thoroughly with PBS and ready to proceed with sensing experiments. *DNA detection protocol*. To assess the performance of the developed DNA sensors, ssDNA target solutions prepared at various concentrations (from 1 to 1000 pM) in 10 mM Tris buffer with 75 mM NaCl, pH 7.5, were incubated on the sensor surface for 15 min. After each incubation step, the biosensor surfaces were thoroughly washed with PBS and transferred to a 2 mM [Fe(CN)_6_]^3–/4–^ solution in 10 mM PBS, where DPV measurements were acquired by scanning the potential from −0.3 to 0.6 V vs Ag/AgCl using an electrochemical analyzer (CH Instruments, model 600D series, USA). To verify the current changes were only caused from the specific hybridization between the immobilized ssDNA capture probe on the carbon-stabilized pSi surface and the incubated target ssDNA, the same measurements were repeated using control biosensors prepared under identical conditions but using random sequences for the ssDNA capture probe. Triplicate measurements were performed with each biosensor.

## Results and Discussion

Scanning electron microscope (SEM) images reveal the morphological features of the TCpSi-THCpSi double-layer structure, in which a TCpSi layer with large (∼80 nm) mesopores rests on top of a THCpSi layer with smaller (∼20 nm) mesopores (Figure 1g–h). The carbonization process (i.e., THCpSi and TCpSi) had a negligible effect on the physical morphology of the individual pSi layers (Figure S1); SEM images showed that single layers prepared with either THCpSi or TCpSi retained their original pSi features (i.e., open pores with similar pore size, shape, and porosity). This was confirmed by optical interferometric reflectance spectroscopy (Figure S2). Previously, Sciacca et al. demonstrated the carbon coating of THCpSi is sufficiently thin to yield adequate optical transparency.^28^ The spectrum of a TCpSi single layer retained the Fabry-Pérot fringes characteristic of the optical interference pattern of a freshly prepared pSi layer. However, the amplitude of the fringes was substantially lower than that measured from the THCpSi samples; this is attributed to the thicker carbon coating of the latter (Figure S2a,b). Ellipsometry analysis showed the carbon layer thickness was 0.72 ± 0.01 and 3.25 ± 0.03 nm for THC- and TC-treated flat Si surfaces, respectively (Table S1). The spectrum of the TCpSi-THCpSi double layer (Figure S2c) shows an interference pattern that combines Fabry-Pérot interferences from both the top and bottom interfaces of each porous layer.^22^

**Figure 2:**
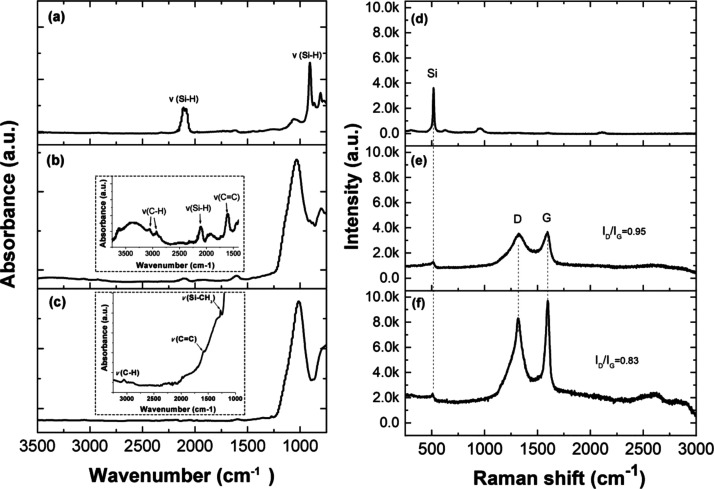
ATR-FTIR spectra of (a) as-etched pSi single layer, (b) TCpSi layer with a pSi bottom layer, and (c) TCpSi-THCpSi double layer. Raman spectra of (d) as-etched pSi single layer, (e) TCpSi single layer, and (f) TCpSi-THCpSi double layer. The intensity ratio of the D and G bands (*I*_D_/*I*_G_) was calculated by Lorentzian fitting of the bands.

Surface chemistry was firstly characterized by means of attenuated total reflectance Fourier transform infrared (ATR-FTIR) spectroscopy. The as-etched pSi layer displayed bands characteristic of Si–H and Si–H_2_ stretching vibrations at 2087 and 2114 cm^–1^, respectively, and a band assigned to the Si–H deformation mode at 905 cm^–1^ (Figure 2a). Compared to the as-etched pSi, TCpSi (Figure S3) did not show the bands characteristic of Si–H; instead new bands associated with the carbon layer were observed: the stretching vibration of saturated C–H at 3047 cm^–1^, the unsaturated carbon double-bond stretching at 1600 cm^–1^, and the CH_3_ symmetric deformation mode of Si–CH_3_ at 1250–1260 cm^–1^.^29^Figure 2b shows the ATR-FTIR spectrum of the TCpSi-pSi double layer, which was derived from an initial TCpSi single layer that was subjected to a second electrochemical anodization process to produce a pSi bottom layer (Figure 1). After the second anodization step, a new band associated with Si–H_*x*_ at ∼2100 cm^–1^ was observed in the TCpSi-pSi double layer. All the bands associated with the original TCpSi layer (e.g., C–H, C=C) persisted in the TCpSi-pSi double layer, suggesting that the carbon-coated layer is stable to the HF-containing electrolyte, and it is not removed by the anodization process. After the second thermal hydrocarbonization step, the resulting TCpSi-THCpSi double layer no longer displayed bands associated with Si–H, while the peaks associated with carbon layers such as C–H, C=C, and CH_3_ were still apparent (Figure 2c).

Raman spectroscopy was used to characterize the carbon layers introduced on the pSi surface. Both the TCpSi single layer (Figure 2e) and the TCpSi-THCpSi double layer (Figure 2f) displayed a characteristic Si lattice mode at 515 cm^–1^ demonstrating that the crystallinity of Si was preserved after carbonization.^30^ Unlike as-etched pSi (Figure 2d), the TCpSi single layer displayed the D and G bands (at 1350 and 1580 cm^–1^, respectively), which are the Raman signatures of carbon materials.^31^ The D band is due to the breathing mode of sp^2^ atoms in rings, and it is linked to defects in the structure. The first-order D band is not usually observed in pristine graphene due to its crystal symmetries.^31^ Hence, the presence of the D band in the Raman spectra of both the TCpSi single layer and the TCpSi-THCpSi double layer suggests the presence of a disordered and defective carbon layer.^32^ In contrast, the G band, primarily an in-plane vibrational mode of sp^2^ atoms in hydrocarbon chains and rings, is indicative of high crystallinity of the carbon layer.^33^ The intensity ratio of the D and G bands (*I*_D_/*I*_G_) is typically used to evaluate the level of disorder in carbon coatings.^34,35^ In the present case, *I*_D_/*I*_G_ values were obtained by Lorentzian fitting of the bands after baseline subtraction. The TCpSi-THCpSi double layer showed a value of *I*_D_/*I*_G_ (0.83) lower than that of the TCpSi single layer (0.95, Figure 2e) suggesting that the deposition and annealing steps (Figure 1) reduced defect density within the carbon coating of the double-layered nanostructure.^36^ To further investigate this characteristic, we calculated *I*_D_/*I*_G_ for a TCpSi-pSi double layer that was prepared from a TCpSi single layer via second anodization but prior to the second carbon deposition step. This sample showed a slightly lower *I*_D_/*I*_G_ value (0.92, Figure S4b) compared with that of the TCpSi single layer precursor (0.95, Figure S4e). This decrease in the level of disordered carbon suggests that the HF-containing electrolyte and the anodization conditions more readily remove the more highly disordered portions of the carbon coating. Thus, the more highly disordered carbon is likely associated with surface SiO_2_, as most of the oxide layer formed on the TCpSi single layer is removed by HF during the second electrochemical etching step. We hypothesize that the greatly reduced defect level in the final TCpSi-THCpSi double-layer structure (evidenced by a lower *I*_D_/*I*_G_ of 0.83, Figure 2f) is due to a combination of increased crystallinity in the bottom layer of THCpSi and the removal of SiO_2_ and disordered carbon from the top layer of TCpSi during the second etching step. The Raman spectra comparing THCpSi and TCpSi single layers with THCpSi-pSi and TCpSi-pSi double layers (Figure S4) confirmed that the carbon coating in both THCpSi and TCpSi is resistant to HF, which is essential to allow the fabrication of multilayered nanostructures that display different degrees of carbonization in one layer relative to the other.

**Figure 3:**
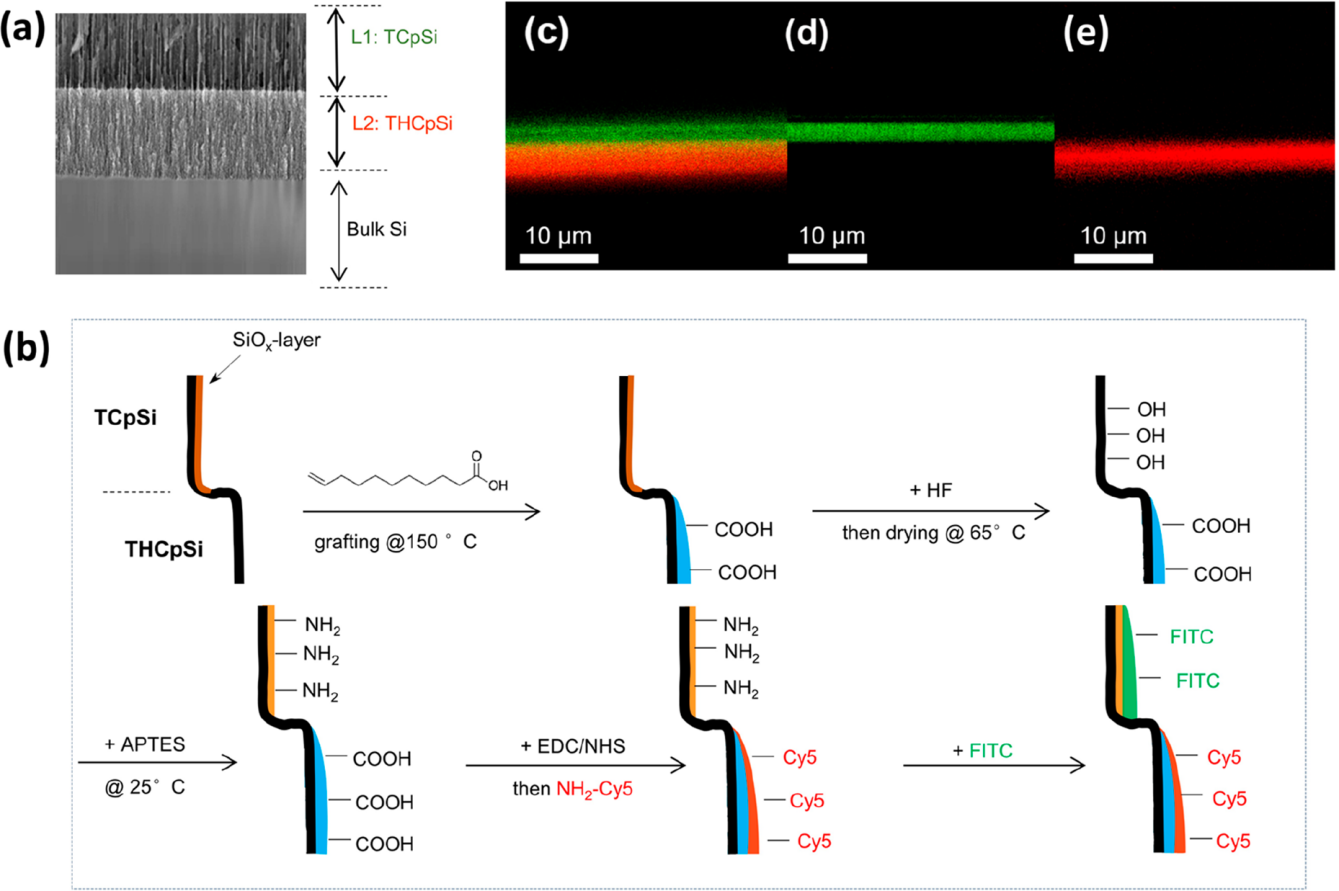
Verification of the selectivity of the chemical functionalization strategy used to modify the TCpSi-THCpSi double-layer nanostructures by laser scanning confocal microscopy. (a) Cross-sectional SEM image revealing the gross pore morphology of the TCpSi-THCpSi double layer: TCpSi top layer (L1) and THCpSi bottom layer (L2); (b) processing scheme followed to prepare the amino and carboxylic acid chemical functionalities on the TCpSi and THCpSi layers, respectively. Also shown is the method used to attach specific green (FITC) and red (Cy5) fluorescent tags to the amino and carboxylic acid moieties, respectively. APTES is 3-aminopropyl triethoxysilane; EDC/NHS is carbodiimide/*N*-hydroxysuccinimide coupling chemistry as described in the text; FITC is fluorescein isothiocyanate; Cy5 is a cyanine dye. (c) Confocal microscope image of the resulting double-layer structure, revealing the distinct FITC-labeled L1 (green) and Cy5-labeled L2 (red) layers, confirming the success of the differential modification strategy; (d) confocal microscope image of control double-layer sample with only FITC-labeled L1; (e) confocal microscope image of control double-layer sample with only Cy5-labeled L2.

We next exploited the controlled surface chemistry of TCpSi-THCpSi to prepare biointerfaces containing different functional groups in the different layers. Amine and carboxylic acid moieties were grafted to the TCpSi and THCpSi surfaces of the double-layer nanostructure, respectively (Figure 3). The site-specific chemistries used to prepare the TCpSi-THCpSi double layer are described in Figure 3b. We first prepared a 7.7 μm-thick TCpSi-THCpSi double layer with the microstructure depicted in Figure 3a. The THCpSi bottom layer was then functionalized with COOH groups via thermal grafting with undecylenic acid.^37^ Next, the TCpSi-THCpSi double layer was treated with aqueous ethanolic HF and dried at 65 °C. While the COOH-terminated THCpSi bottom layer was stable to HF, the TCpSi top layer reacted with the HF-containing solution to introduce surface −OH groups.^25^ This reactivity was confirmed by performing the same HF treatment on a TCpSi single layer; the ATR-FTIR data are shown in Figure S5, which is consistent with previous reports.^25^ A broadening of the OH band over 3000 cm^–1^ and a clear shoulder structure around 3650 cm^–1^ is indicative of the formation of OH groups (Figure S5b). The TCpSi surface, while primarily composed of silicon and carbon species, also contained some surface oxide species (primarily Si oxide that is back-bonded to carbon atoms);^36^ exposure of this surface to HF generated the surface hydroxyls. The hydroxylated TCpSi top layer was then functionalized with 3-aminopropyl triethoxysilane (APTES), a common linker used to graft amines to silanol or hydroxyl surfaces. This resulted in an amine-terminated TCpSi top layer.

The two layers were then differentially modified with fluorescent dyes in order to validate the differential grafting chemistries. The COOH species on the THCpSi bottom layer was attached to the free NH_2_ group on a Cy5 fluorescent dye via carbodiimide coupling chemistry, and the fluorescent dye fluorescein was grafted to the NH_2_-terminated TCpSi top layer by reaction with fluorescein isothiocyanate (FITC). The site-specific labeling of the TCpSi-THCpSi double layer was confirmed by confocal fluorescence microscopy. As presented in Figure 3c, the green and red layers correspond to the FITC-labeled TCpSi top layer and Cy5-labeled THCpSi bottom layer, respectively. Control confocal microscope images were acquired on TCpSi-THCpSi double-layer samples that were functionalized exclusively with either FITC or Cy5. Functionalized TCpSi-THCpSi double-layer samples labeled with FITC showed characteristic green fluorescence only at the TCpSi top layer (Figure 3d), while samples labeled with Cy5 displayed red fluorescence only at the THCpSi bottom layer (Figure 3e). The thickness and location of each dye-labeled layer were consistent with the physical thickness and location of the layers as measured by SEM (Figure S6).

**Figure 4:**
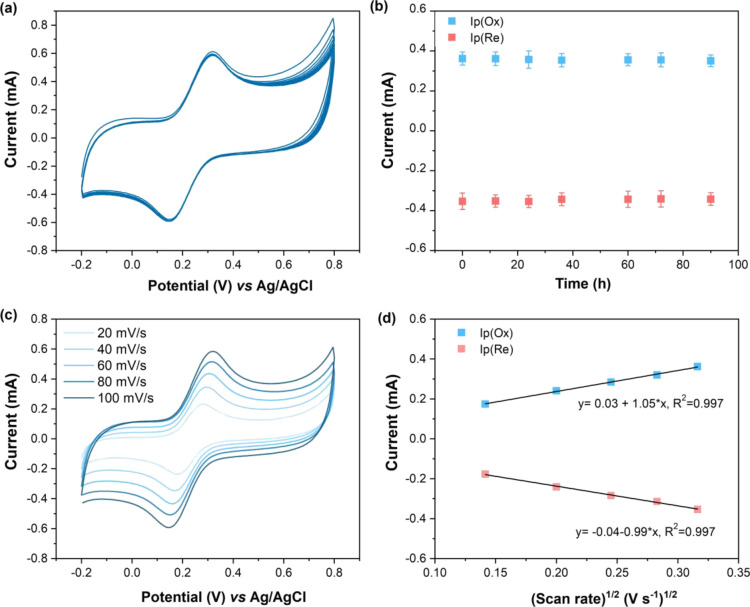
Electrochemical characterization of the TCpSi-THCpSi double-layer nanostructure in a 2 mM [Fe(CN)_6_]^3–/4–^ solution in 10 mM PBS, pH 7.4. (a) 10 cyclic voltammograms obtained by sweeping the potential from −0.2 to 0.8 V vs Ag/AgCl at a scan rate of 0.1 V s^–1^; (b) oxidation (*I*_p_(Ox)) and reduction (*I*_p_(Re)) current intensity values vs time, obtained from cyclic voltammograms measured under the same conditions as in (a); (c) cyclic voltammograms obtained by sweeping the potential from −0.2 to 0.8 V vs Ag/AgCl at scan rates ranging from 0.02 to 0.1 V s^–1^; (d) plot of *I*_p_(Ox) and *I*_p_(Re) extracted from the cyclic voltammograms in (c) vs ν^1/2^.

The electrochemical properties of the TCpSi-THCpSi double-layer nanostructure were investigated by cyclic voltammetry (CV) in the presence of [Fe(CN)_6_]^3–/4–^ in 10 mM phosphate-buffered saline solution (PBS), at pH 7.4. The [Fe(CN)_6_]^3–/4–^ redox system, extensively used in the electrochemical characterization of sp^2^ carbon materials (e.g., graphite and glassy carbon), was chosen because it displays quasi-reversible redox electrochemistry on carbon electrodes.^38^ First, to demonstrate the ability of the whole double-layer nanostructure to work as an electrochemical transducer, the effective surface area (*A*_eff_) of both TCpSi single layer and TCpSi-THCpSi double-layer, the latter with a top TCpSi featuring the same morphology as the single layer, was estimated from the electrochemical data obtained from cyclic voltammograms in a 2 mM [Fe(CN)_6_]^3–/4–^ solution, by using the Randles–Sevick equation. The *A*_eff_ of the TCpSi single layer was 0.61 cm^2^, a value that increased to 0.96 cm^2^ for the TCpSi-THCpSi double layer, confirming the accessibility of the bottom porous layer and the effectiveness of the whole structure to work as an electrochemical transducer. The TCpSi-THCpSi double-layer structure showed reasonable performance as an electrode for redox events in the range from −0.2 to 0.8 V vs Ag/AgCl, as shown by the high oxidation and reduction peak currents and small peak-to-peak potential difference Δ*E*_p_ (Figure 4a). The Δ*E*_p_ value of 123 mV is larger than the expected theoretical value of 59 mV at 25 °C for a fully reversible one-electron transfer reaction, representing a quasi-reversible electron-transfer system.^39,40^ The ratio of peak currents |*I*_p_(Ox)|/|*I*_p_(Re)| was approximately 1 (Table S2), indicating that the stabilized TCpSi-THCpSi double-layer nanostructure possessed surface and electronic properties sufficient to support relatively rapid electron transfer. The fast electron transfer these nanostructures elicit was confirmed by the estimated value of heterogeneous standard rate constant *k*_0_ of electron transfer. We calculated *k*_0_ using a modified version of the Nicholson method reported by Lavagnini and colleagues^41^ that introduces an empirical equation to simplify the calculation of the dimensionless parameter, Ψ:
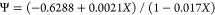
where *X* is Δ*E*_p_ in mV (Δ*E*_p_ values extracted from Figure 4c are included in Table S2). The relationship between Ψ and *k*_0_ is given by the following equation:
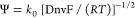


Therefore, the slope of the linear fitting of the plot Ψ vs [π*DnF*/(*RT*)]^−1/2^ ν^–1/2^ can be assigned to the kinetic parameter *k*_0_, which corresponded to 0.16 cm s^–1^ for the carbon-stabilized TCpSi-THCpSi double-layer nanostructure. This value exceeds the *k*_0_ value of 3.6 × 10^–4^ cm s^–1^, reported by Lavagnini et al.^41^ for a carbon paste electrode under the same working conditions. It also supports fast electron transfer when compared with a glassy carbon electrode, based on the *k*_0_ values estimated using other methods of calculation: 8.9 × 10^–3^ cm s^–1^ determined by digital simulation,^42^ or 1.3 × 10^–3^ cm s^–1^ estimated from EIS data.^43^

The stability of the electrode was evaluated by recording |*I*_p_(Ox)| and |*I*_p_(Re)| values through many cycles, over a time period of 90 h. The response was stable over this period, with average peak current values of 0.36 ± 0.01 and 0.35 ± 0.01 mA, respectively (RSD < 3%) (Figure 4b). Moreover, *I*_p_(Ox) and *I*_p_(Re) increased linearly with the square root of scan rate ν^1/2^ (Figure 4c,d), suggesting that even in the restricted dimensions of the porous nanostructure, the reaction is controlled by semi-infinite linear diffusion typical of planar electrodes at the timescales measured.^44^

To demonstrate the suitability of the TCpSi-THCpSi double-layer structures for electrochemical biosensing, a label-free voltammetric DNA sensor was constructed. The TCpSi top layer, possessing relatively large pores of 27 ± 9 nm diameter, was selectively functionalized with an ssDNA probe while the THCpSi bottom layer with smaller pores (<5 nm diameter) acted as an electrochemical transducer to convert the hybridization event confined in the top layer into an output current signal via differential pulse voltammetry (DPV) measurements. Successful immobilization of the ssDNA probe on the top layer of the TCpSi-THCpSi biosensor was confirmed by ATR-FTIR spectroscopy at each modification step (Figure S7).

**Figure 5:**
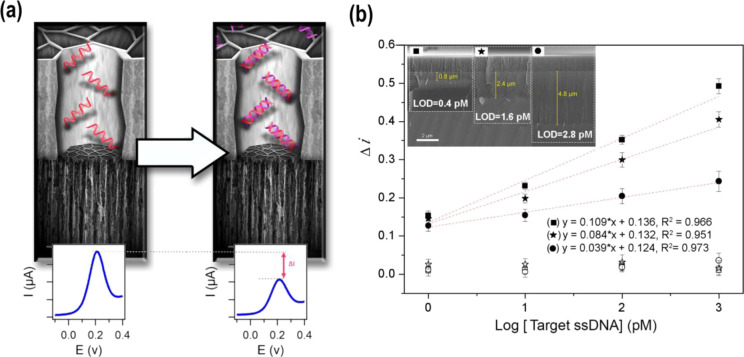
(a) Schematic of the sensing mechanism of the voltammetric DNA sensor designed using a TCpSi-THCpSi double-layer nanostructure. The biosensor measures the electrochemical current of a ferricyanide/ferrocyanide redox couple at the electrochemically active bottom layer of the structure. The top layer, containing an ssDNA capture probe chemically grafted to the inner pore walls, modulates this current by impeding transport of the redox couple when the complementary ssDNA analyte is hybridized. (b) Dose–response curves for the detection of a 28-nucleotide ssDNA in Tris buffer using TCpSi-THCpSi double layer-based biosensors prepared with either specific ssDNA capture probes (solid symbols) or nonspecific ssDNA capture probes as controls (hollow symbols). Data are shown as the mean of normalized current intensity ± standard deviation, *n* = 3. DPV measurements were performed in a 2 mM [Fe(CN)_6_]^3–/4–^ solution in 10 mM PBS, pH 7.4. Inset: Cross-sectional SEM images of the double layer; TCpSi top recognition layer with an average pore size of 27 ± 9 nm, and 1.6 μm in depth; THCpSi bottom transducer layer with small pores (<5 nm diameter), and various depths (0.8, 2.4, and 4.8 μm).

The sensing mechanism of the label-free voltammetric DNA sensor developed here relies on the hypothesis that DNA hybridization will induce partial blockage of the nanochannels as shown in Figure 5a. When the target ssDNA hybridizes with the ssDNA capture probe immobilized at the TCpSi top layer, partial blockage of the top layer nanochannels can be expected. This partial nanochannel blockage hinders the diffusion of redox species such as [Fe(CN)_6_]^3–/4–^ into the electrochemically active bottom layer (the electrochemical transducer), resulting in a decrease in the intensity of peak current monitored by DPV. Changes in peak current obtained from differential pulse voltammograms acquired prior to and after hybridization were normalized via the relationship:
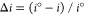
1where Δ*i* is the normalized current change, *i°* is the peak current value measured after 15 min incubation in buffer blank, and *i* is the peak current value measured after 15 min incubation in the ssDNA target solution at a given concentration. Both *i°* and *i* were extracted from the differential pulse voltammograms obtained in a 2 mM [Fe(CN)_6_]^3–/4–^ solution. Control samples were prepared using a random sequence of ssDNA as a capture probe.

Three double-layer structure types were tested: all three were prepared with a top layer of similar thickness and porosity, and the thickness of the bottom layer (the transducer layer) was systematically varied, and the analytical performance of each DNA sensor was assessed. The normalized Δ*i* values plotted as a function of Log[ssDNA] (Figure 5) exhibited a linear relationship to the target ssDNA concentration in the range from 1 to 1000 pM. Control experiments using a noncomplementary capture probe (hollow symbols) showed nearly no electrochemical response compared with the corresponding specific DNA sensors (solid symbols). As shown in Figure 5, the transducer thickness (i.e., THCpSi bottom layer) had a pronounced effect on the sensitivity of the designed DNA sensor. The structure with the thinnest THCpSi bottom layer tested (0.8 μm) showed the highest sensitivity.

The LOD values, calculated using the equation *y*_b_ + 3 × SD, where *y*_b_ is the DPV current value for the blank (normalized Δ*i* in buffer) and SD is the associated standard deviation (*n* = 3), were 0.4, 1.6, and 2.8 pM for TCpSi-THCpSi double-layer nanostructured DNA sensors featuring 0.8, 2.4, and 4.8 μm transducer thickness at the bottom layer, respectively. The developed pSi double layer-based DNA sensor allowed the detection of a 28-nucleotide DNA sequence, with a LOD as low as 0.4 pM, showing two orders of magnitude enhancement compared with the best LOD (50 pM)^23^ achieved to date by a pSi-based electrochemical DNA sensor reported by Lugo et al., which exploited the semiconductor characteristics of pSi. That DNA sensor used oxidized pSi as the immobilization platform of a DNA capture probe complementary to the target DNA, and DNA hybridization was monitored by measuring the oxidation of guanine using a ruthenium bipyridine [Ru(bpy)_3_]^2+^ redox indicator.^23^ The present approach takes advantage of the electrical conductivity of the carbonized pSi matrix, allowing for a more stable and more sensitive detection modality.

For electrochemical sensing, the double-layer structure shows a key advantage over a single-layer structure because it provides a robust means to incorporate an electrochemical transducer into the nanostructured sensing element. For example, when a single layer-based TCpSi transducer is used, the biomolecular coating acts as a highly resistive series element, inhibiting electron transfer from solution species into the electrode and thus degrading its performance as an electrochemical transducer. The electrochemical characterization data obtained on such a control single-layer structure is given in Table S3. Covalent immobilization of the capture probe (ssDNA) to a TCpSi single layer caused a sharp decrease in the measured anodic and cathodic peak currents, and an increase in Δ*E*_p_ in the cyclic voltammograms. The inclusion of an unmodified THCpSi layer featuring small (<5 nm) pores directly underneath the DNA-modified TCpSi layer overcomes this limitation; indeed, the electrochemical performance exceeded the sensitivity achieved on conventional flat electrodes.^45−49^ Because the pore size and thickness of THCpSi bottom layer are readily adjustable in the fabrication process, the double-layer system provides a means to optimize surface area of the transducer, as well as its wettability. The small dimension of the micropores in this electrochemically active layer also acts to exclude larger species (such as proteins) that might interfere with the electrochemical measurement. Compared to single layer-based transducers, TCpSi-THCpSi double layers also offer the possibility to tune the electrochemical performance by varying pore size and depth in each layer, providing a means to tune the sensitivity level required for a specific biosensing purpose.

## Conclusions

In summary, this work demonstrates for the first time the design and fabrication of a carbon-stabilized pSi double-layer nanostructure featuring fine-tuned wettability and layer-specific functionalization to facilitate further fit-for-purpose modification. Along with retaining the unique physical features derived from pSi, the carbon-stabilized TCpSi-THCpSi double-layer nanostructures possess controllable surface functionalities and fast electron transfer kinetics suitable to harness them as both a biointerface and an electrochemical transducer. Critical to designing high-performance electrochemical biosensors is the demonstrated capacity of the carbon-stabilized TCpSi-THCpSi double-layer nanostructures to tune their sensitivity to suit to a specific application. Our results support the feasibility of increasing sensitivity by adjusting the morphological features of the transducer layer to maximize the electrode active area and those of the biorecognition layer to maximize pore blockage upon analyte binding. Their potential for use as electrochemical biosensors is demonstrated by the developed TCpSi-THCpSi double-layer based voltammetric DNA sensor, showing an LOD of 0.4 pM in buffer, two orders of magnitude lower than the best performing previously reported pSi-based electrochemical DNA sensor. While not as sensitive as the best label-free electrochemical biosensors^49^ or PCR methods that can detect DNA in the low fM range,^50^ the controllability of the nanostructural morphology and surface chemistry of this combination of carbon and pSi provides a means to incorporate features, such as selective filtration, that can enable more complex (bio)sensing functions. Future research will unlock the potential of these double-layer structures as sensing platforms for sequential detection of various analytes. Sequential sensing is envisaged by combining discrimination based on molecular affinity, provided by the site-specifically immobilized bioreceptors and the differential penetration of molecules into specific layers.

## Supporting Information Available

SEM images of pSi, THCpSi, and TCpSi; Interferometric reflectance spectra of pSi, TCpSi, and TCpSi-THCpSi; attenuated total reflectance Fourier transform infrared (ATR-FTIR) spectrum of TCpSi; Raman spectra of THCpSi-pSi, TCpSi-pSi, pSi, THCpSi, and TCpSi; ATR-FTIR spectra of TCpSi before and after HF immersion; cross-sectional SEM image of the pSi double-layer sample used for confocal microscopy characterization after selective dye labeling; ATR-FTIR spectra of TCpSi-THCpSi after HF-dipping and drying at 65 °C for 3 h, epoxy silane modification, and NH_2_-ssDNA probe immobilization; ellipsometry analysis to calculate the thickness of carbon layer; summary of peak current and Δ*E*_p_ extracted from the cyclic voltammograms obtained using TCpSi-THCpSi; summary of peak currents and Δ*E*_p_ extracted from the cyclic voltammograms obtained during functionalization and DNA immobilization, using TCpSi, and TCpSi-THCpSi (PDF)
