# Supplementary material for: Designing Electrochemical Biosensing Platforms Using Layered Carbon-Stabilized Porous Silicon Nanostructures
Source: ACS Appl Mater Interfaces. 2022 Mar 14;14(13):15565–75. doi: 10.1021/acsami.2c02113 (PMC9682479; doi:10.1021/acsami.2c02113)
Supplement: Supplementary file 1 — am2c02113_si_001.pdf [file am2c02113_si_001.pdf]

Supporting Information

Designing Electrochemical Biosensing Platforms Using Layered Carbon-Stabilized Porous Silicon Nanostructures

Keying Guo[1,2#], Maria Alba[1,2,3], Grace Pei Chin[1], Ziqiu Tong[1], Bin Guan[4], Michael J. Sailor[5], Nicolas H. Voelcker[1,2,3,*], and Beatriz Prieto-Simón[6,7,*]

[1] Monash Institute of Pharmaceutical Sciences, Monash University, Parkville, Victoria, 3052, Australia
[2] Melbourne Centre for Nanofabrication, Victorian Node of the Australian National Fabrication Facility, Clayton, Victoria, 3168, Australia
[3] Commonwealth Scientific and Industrial Research Organisation (CSIRO), Clayton, Victoria, 3168, Australia
[4] Future Industries Institute, University of South Australia, Mawson Lakes, South Australia, 5095, Australia
[5] Department of Chemistry and Biochemistry and Department of Nanoengineering, University of California, San Diego, La Jolla, California, 92093-0358, United States
[6] Department of Electronic Engineering, Universitat Rovira i Virgili, 43007, Tarragona, Spain
[7] ICREA, Pg. Lluís Companys 23, 08010, Barcelona, Spain

*Email: nicolas.voelcker@monash.edu; beatriz.prieto-simon@urv.cat

Present Address
King Abdullah University of Science and Technology (KAUST), Biological and Environmental Science and Engineering (BESE), Thuwal, 23955-6900, Saudi Arabia

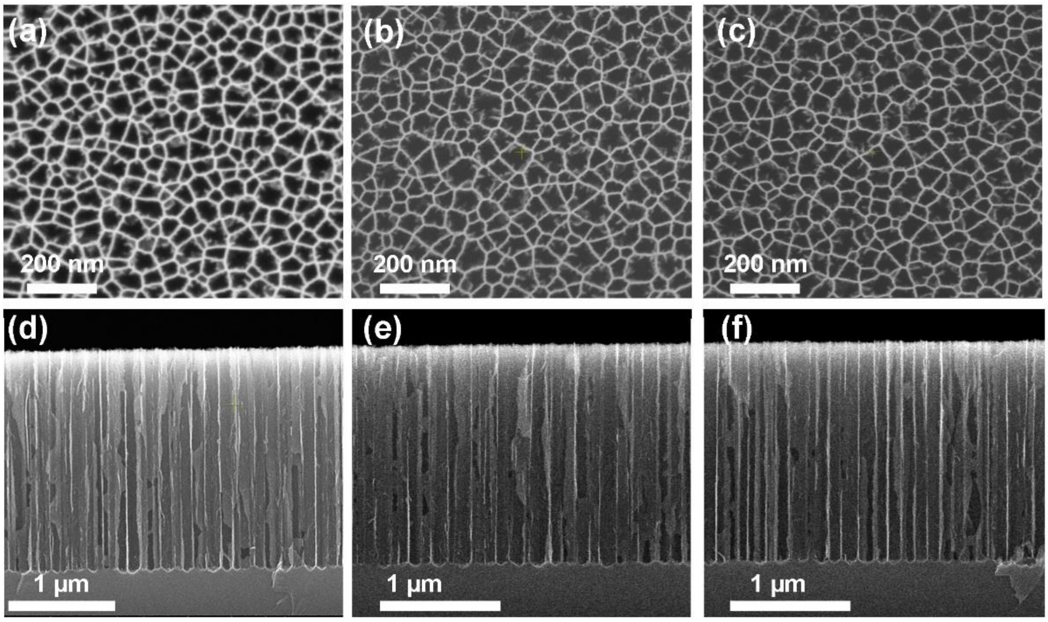

Figure S1. SEM images of pSi single layer structures fabricated using a wet etching system (A.M.M.T GmbH, Germany) with a current density of 65 mA cm^{-2} in 1:1 (v:v) aqueous 48% HF:absolute ethanol electrolyte for 90 s: top views of (a) pSi, (b) THCpSi and (c) TCpSi structures; and corresponding cross-sectional views of (d) pSi, (e) THCpSi and (f) TCpSi structures. Pore size and film thickness are 79 ± 19 nm and 1.8 μm, respectively.

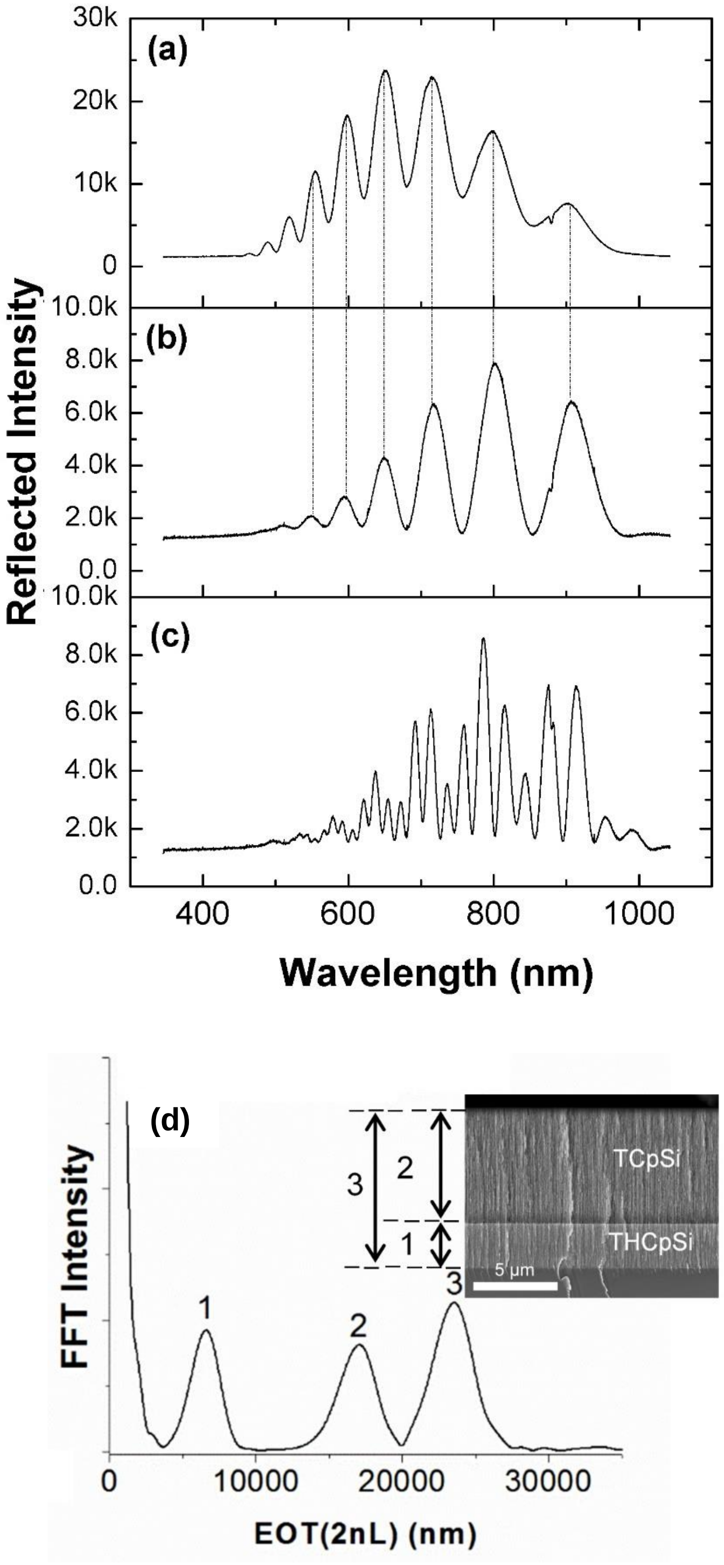

Figure S2. Interferometric reflectance spectra of (a) as-etched pSi single layer, (b) TCpSi single layer, and (c) TCpSi-THCpSi double layer. The top pSi layer was fabricated using a small etching cell with a current density of 50 mA cm^{-2} in 1:1 (v:v) aqueous 48% HF:absolute ethanol solution for 300 s, followed by thermal carbonization to generate TCpSi, as described in the text. The sample containing the TCpSi top layer was then subjected to a second etch using the same small etching cell with a current density of 67 mA cm^{-2} in 3:1 (v:v) aqueous

48% HF:absolute ethanol solution for 120 s. The thicknesses of the TCpSi top layer and THCpSi bottom layer were 7.3 and 2.8 µm, respectively. (d) Corresponding fast Fourier transform (FFT) of the interferometric reflectance spectrum shown in (c). Inset of (d) shows a representative cross-sectional SEM image of the final TCpSi-THCpSi sample.

Fringe maxima follow the Fabry-Pérot relationship:[1]

$$m\lambda = 2nL \qquad (1)$$

where *m* is an integer, λ is the wavelength of incident light, *n* is the average refractive index of the porous matrix (including the contents of the pores), and L is the physical thickness of the pSi layer. The light reflected from the double-layer structure comprises three superimposed interference patterns. The analysis of the reflectance spectra can be simplified by transforming reflectance oscillations into three peaks *via* FFT as described previously.[2] The value of *2nL* of each porous layer can be directly interpreted as the position of the FFT peaks (**Figure S2**d). The double-layer film in air produces peak 1 at 6580 nm, peak 2 at 17072 nm, and peak 3 at 23652 nm, corresponding to the values of *2nL* for bottom layer 1, top layer 2 and double layer 3, respectively. The effective optical thickness (EOT) of each layer, equivalent to *2nL,* can be used to measure changes in refractive index assuming constant thickness, thus providing a simple and effective approach to monitor changes in complex optical structures.[2]

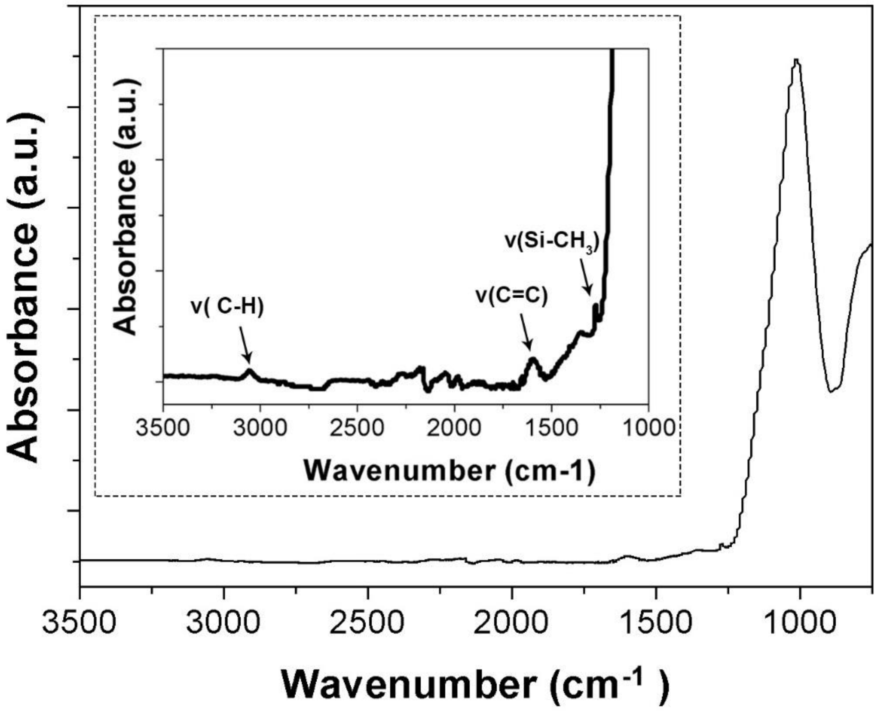

Figure S3. Attenuated total reflectance Fourier transform infrared (ATR-FTIR) spectrum of TCpSi single layer.

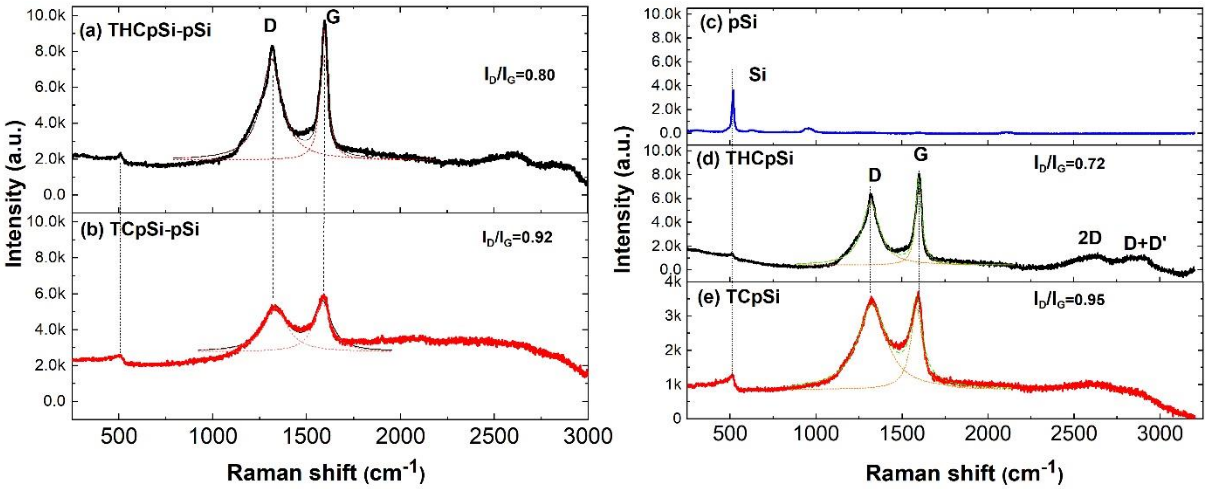

Figure S4. Raman spectra of (a) THCpSi-pSi and (b) TCpSi-pSi double layers, and (c) as-etched pSi, (d) THCpSi and (e) TCpSi single layers. The peaks at ~1350 and ~1580 cm^{-1} are attributed to the D and G bands of carbon, respectively. The intensity ratio of the D and G bands (I_D/I_G) was calculated from a Lorentzian fit of the data.

Results in **Figure S4** demonstrate the carbon coatings in both THCpSi and TCpSi structures are resistant to HF, enabling the fabrication of multilayered nanostructures. Both THCpSi and TCpSi single layers were subjected to a second electrochemical etching step in 3:1 HF/ethanol etchant, providing THCpSi-pSi and TCpSi-pSi double layers with a freshly etched pSi bottom layer. Their Raman spectra **(Figure S4** a-b**)** and those of both THCpSi and TCpSi single layers (**Figure S4** d-e) are shown. **Figure S4** a-b shows that the carbon D and G bands of both THCpSi and TCpSi structures are retained upon etching of the pSi bottom layer.

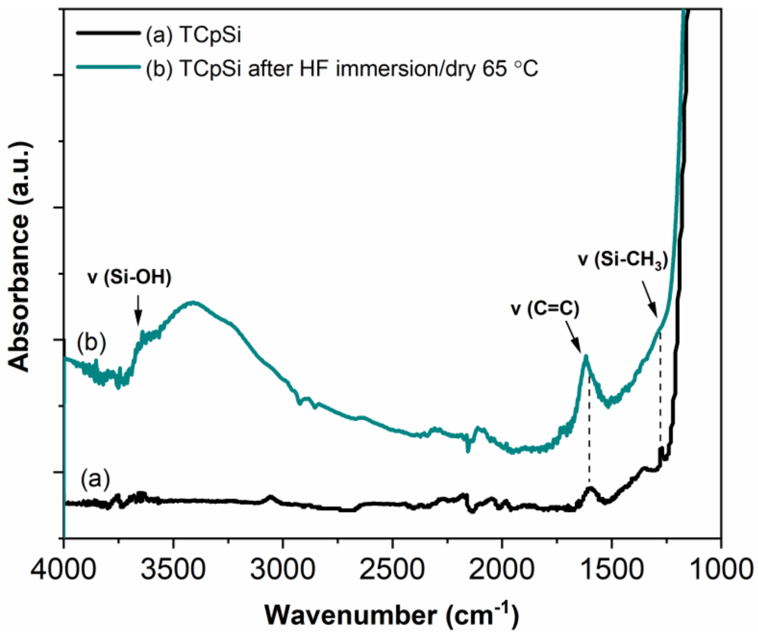

Figure S5. ATR-FTIR spectra of TCpSi single layer (a) before and (b) after HF immersion. The same TCpSi single layer from Figure S3 was immersed into 1:1 (v:v) aqueous 48% HF:absolute ethanol solution for 15 min at room temperature, then removed from the solution, rinsed with absolute ethanol, and finally dried at 65°C for 3 h.

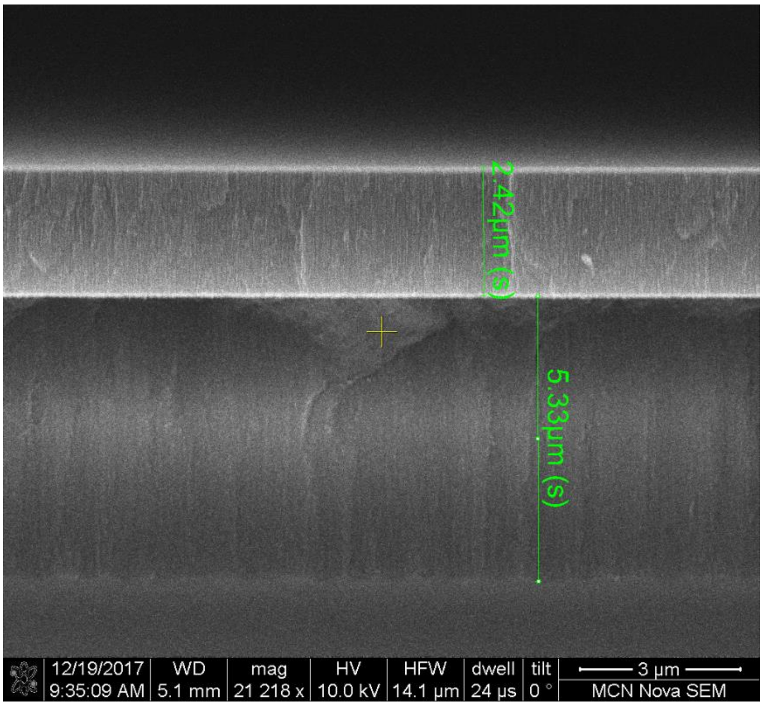

Figure S6. Cross-sectional SEM image of the pSi double-layer sample used for confocal microscopy characterization after selective dye labeling. The thicknesses of the top and bottom porous layers are approximately 2.4 and 5.3 µm, respectively. The pSi top layer was electrochemically etched in a 1:1 (v:v) aqueous 48% HF:absolute ethanol solution, by applying a current density of 12.1 mA cm^{-2} for 360 s in a wet electrochemical etching system (A.M.M.T GmbH, Germany), while the pSi bottom layer was etched using a small cell in 3:1 (v:v) aqueous 48% HF:absolute ethanol with a current density of 83.3 mA cm^{-2} applied for 180 s.

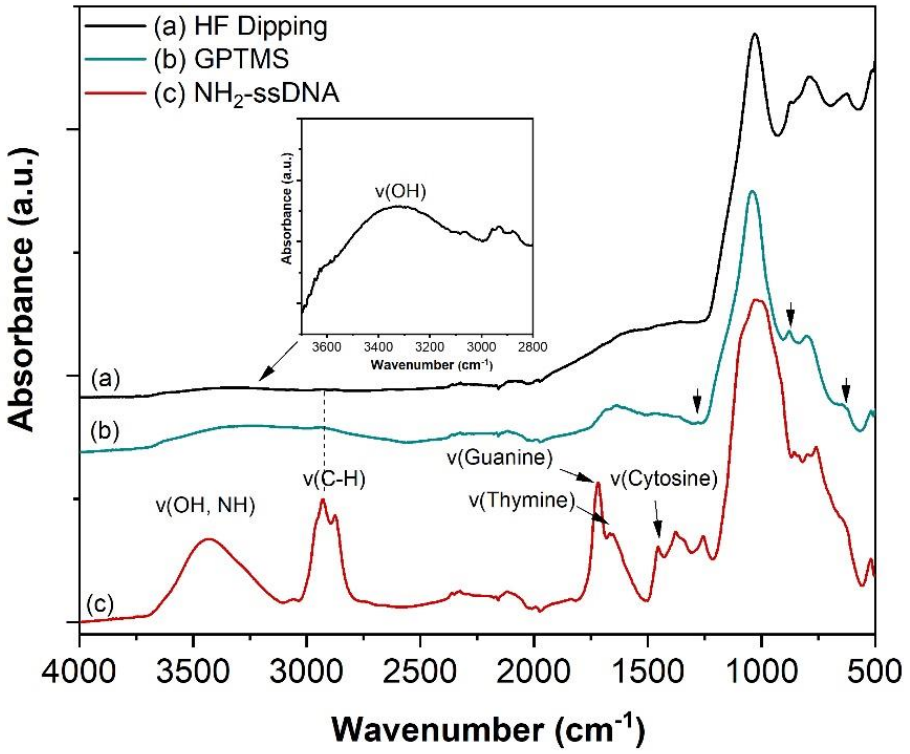

Figure S7. ATR-FTIR spectra of a TCpSi-THCpSi double layer after (a) HF-dipping and drying at 65°C for 3 h, (b) epoxy silane modification and (c) NH_2-ssDNA probe immobilization.

Epoxide groups grafted to the OH-terminated TCpSi top layer via silanization with (3-glycidyloxypropyl) trimethoxysilane (GPTMS) allowed further covalent binding of NH_2-terminated ssDNA probes. These surface modification steps were characterized by ATR-FTIR spectroscopy, **Figure S7** depicting the relevant spectra. TCpSi-THCpSi double layer was treated in 1:1 (v:v) HF:ethanol solution for 15 min and dried at 65°C for 3 h. As shown in **Figure S7**a, the broad band at 3500 cm^{-1} is assigned to O-H stretching. This is consistent with previous work by Salonen *et al.*, who reported that exposure of the TCpSi surface to HF enables its hydroxylation.[3] After modification with GPTMS, the FTIR spectrum displayed bands at 632, 880, and 1277 cm^{-1} which are assigned to epoxide ring vibrations (**Figure S7**b).[4] Covalent binding of the ssDNA probe led to several new peaks depicted in **Figure S7**c. Bands characteristic of DNA bases were observed at 1456 cm^{-1} (cytosine), 1653 cm^{-1} (thymine), and 1713 cm^{-1} (guanine).[5, 6] Bands at 1255 and 1374 cm^{-1} are assigned to antisymmetric stretching modes of the phosphate backbone groups.[5]

Table S1. Ellipsometry analysis to calculate the thickness of the carbon layer on various carbonized substrates. A flat Si surface was either THC or TC treated following the protocol previously described in the section *Thermal carbonization of pSi single layer*. For the control sample (HF rinsed Si surface, noted as Si/HF), a native oxide layer with a thickness of 0.69 (± 0.02) nm was observed. The thickness of such native oxide layer was considered for the analysis of the THC-treated flat Si surface (THC-Si), TC-treated flat Si surface (TC-Si) and HF-rinsed samples (THC-Si/HF, TC-Si/HF).

**Sample**	**Thickness of carbon layer (nm)**
TC-Si	3.36 ± 0.03
TC-Si/HF	3.25 ± 0.03
THC-Si	0.78 ± 0.01
THC-Si/HF	0.72 ± 0.01

Table S2. Summary of peak current (|Ip(Ox)| and |Ip(Re)|) and $\triangle E_p$ extracted from the cyclic voltammograms obtained using TCpSi-THCpSi double layer structures in a 2 mM $[Fe(CN)_6]^{3-/4-}$ in 10 mM PBS, pH 7.4, by sweeping the potential from -0.2 to 0.8 V vs Ag/AgCl, at various scan rates.

**Scan rate (V s^{-1})**	**|Ip(Ox)| (mA)**	**|Ip(Re)| (mA)**	**|Ip(Ox)|/|Ip(Re)|**	**$\triangle E_p$ (mV)**
0.02	0.174	0.177	0.98	101
0.04	0.241	0.239	1.01	129
0.06	0.285	0.284	1.01	143
0.08	0.321	0.314	1.02	166
0.10	0.362	0.353	1.03	175

Table S3. Summary of peak current (|Ip(Ox)|, |Ip(Re)|) and $\triangle E_p$ extracted from the cyclic voltammograms obtained during functionalization and DNA immobilization, using TCpSi single-layer and TCpSi-THCpSi double-layer structures. Measurements were performed in a 2 mM $[Fe(CN)_6]^{3-/4-}$ solution prepared in 10 mM PBS, pH 7.4, in a potential range from -0.2 to 0.8 V vs Ag/AgCl, at a scan rate of 0.1 V/s. The pSi top layer was electrochemically etched in a 1:1 (v:v) aqueous 48% HF:absolute ethanol solution, by applying a current density of 18.9 mA cm^{-2} for 80 s in a wet etching system (A.M.M.T GmbH, Germany), while the pSi bottom layer was etched using a small cell in 3:1 (v:v) aqueous 48% HF:absolute ethanol with a current

density of 21.2 mA cm^{-2} applied for 30, 60 and 150 s to produce thicknesses of 0.4, 0.8 and 2.4 µm, respectively.

<table>
<tr><th colspan="2">Type of platform</th><th>Surface modification</th><th>|Ip(Ox)| (mA)</th><th>|Ip(Re)| (mA)</th><th>$\triangle E_p$ (mV)</th></tr>
<tr><td colspan="2" rowspan="2">TCpSi single layer</td><td>+ GTPMS</td><td>0.426</td><td>0.43</td><td>130</td></tr>
<tr><td>+ ssDNA</td><td>0.361</td><td>0.34</td><td>160</td></tr>
<tr><td rowspan="7">TCpSi-THCpSi double layer</td><td>Bottom layer thickness (µm)</td><td></td><td></td><td></td><td></td></tr>
<tr><td rowspan="2">0.4</td><td>+ GTPMS</td><td>0.445</td><td>0.454</td><td>125</td></tr>
<tr><td>+ ssDNA</td><td>0.436</td><td>0.421</td><td>128</td></tr>
<tr><td rowspan="2">0.8</td><td>+ GTPMS</td><td>0.433</td><td>0.436</td><td>125</td></tr>
<tr><td>+ ssDNA</td><td>0.433</td><td>0.435</td><td>125</td></tr>
<tr><td rowspan="2">2.4</td><td>+ GTPMS</td><td>0.425</td><td>0.384</td><td>135</td></tr>
<tr><td>+ ssDNA</td><td>0.421</td><td>0.418</td><td>140</td></tr>
</table>

References

(1) Pacholski, C.; Sartor, M.; Sailor, M. J.; Cunin, F.; Miskelly, G. M. Biosensing Using Porous Silicon Double-Layer Interferometers: Reflective Interferometric Fourier Transform Spectroscopy. *J. Am. Chem. Soc.* **2005**, *127* (33), 11636-11645.

(2) Orosco, M. M.; Pacholski, C.; Sailor, M. J. Real-Time Monitoring of Enzyme Activity in a Mesoporous Silicon Double Layer. *Nat. Nanotechnol.* **2009**, *4* (4), 255-258.

(3) Makila, E.; Bimbo, L. M.; Kaasalainen, M.; Herranz, B.; Airaksinen, A. J.; Heinonen, M.; Kukk, E.; Hirvonen, J.; Santos, H. A.; Salonen, J. Amine Modification of Thermally Carbonized Porous Silicon with Silane Coupling Chemistry. *Langmuir* **2012**, *28* (39), 14045-14054.

(4) Tiringer, U.; Milošev, I.; Durán, A.; Castro, Y. Hybrid Sol–Gel Coatings Based on Gptms/Teos Containing Colloidal Sio 2 and Cerium Nitrate for Increasing Corrosion Protection of Aluminium Alloy 7075-T6. *Journal of Sol-Gel Science and Technology* **2018**, *85* (3), 546-557.

(5) Cui, L.-Y.; Fang, X.-H.; Cao, W.; Zeng, R.-C.; Li, S.-Q.; Chen, X.-B.; Zou, Y.-H.; Guan, S.-K.; Han, E.-H. In Vitro Corrosion Resistance of a Layer-by-Layer Assembled DNA Coating on Magnesium Alloy. *Appl. Surf. Sci.* **2018**, *457*, 49-58.

(6) van den Beucken, J. J.; Vos, M. R.; Thüne, P. C.; Hayakawa, T.; Fukushima, T.; Okahata, Y.; Walboomers, X. F.; Sommerdijk, N. A.; Nolte, R. J.; Jansen, J. A. Fabrication, Characterization, and Biological Assessment of Multilayered DNA-Coatings for Biomaterial Purposes. *Biomaterials* **2006**, *27* (5), 691-701.
